# Dance Movements Enhance Song Learning in Deaf Children with Cochlear Implants

**DOI:** 10.3389/fpsyg.2016.00835

**Published:** 2016-06-15

**Authors:** Tara Vongpaisal, Daniela Caruso, Zhicheng Yuan

**Affiliations:** Department of Psychology, MacEwan UniversityEdmonton, AB, Canada

**Keywords:** auditory-motor learning, multimodal learning, music, dance, deafness, cochlear implants, children

## Abstract

Music perception of cochlear implants (CI) users is constrained by the absence of salient musical pitch cues crucial for melody identification, but is made possible by timing cues that are largely preserved by current devices. While musical timing cues, including beats and rhythms, are a potential route to music learning, it is not known what extent they are perceptible to CI users in complex sound scenes, especially when pitch and timbral features can co-occur and obscure these musical features. The task at hand, then, becomes one of optimizing the available timing cues for young CI users by exploring ways that they might be perceived and encoded simultaneously across multiple modalities. Accordingly, we examined whether training tasks that engage active music listening through dance might enhance the song identification skills of deaf children with CIs. Nine CI children learned new songs in two training conditions: (a) listening only (auditory learning), and (2) listening and dancing (auditory-motor learning). We examined children's ability to identify original song excerpts, as well as mistuned, and piano versions from a closed-set task. While CI children were less accurate than their normal hearing peers, they showed greater song identification accuracies in versions that preserved the original instrumental beats following learning that engaged active listening with dance. The observed performance advantage is further qualified by a medium effect size, indicating that the gains afforded by auditory-motor learning are practically meaningful. Furthermore, kinematic analyses of body movements showed that CI children synchronized to temporal structures in music in a manner that was comparable to normal hearing age-matched peers. Our findings are the first to indicate that input from CI devices enables good auditory-motor integration of timing cues in child CI users for the purposes of listening and dancing to music. Beyond the heightened arousal from active engagement with music, our findings indicate that a more robust representation or memory of musical timing features was made possible by multimodal processing. Methods that encourage CI children to entrain, or track musical timing with body movements, may be particularly effective in consolidating musical knowledge than methods that engage listening only.

## Introduction

When asked to explain how she attains the elusive musicality in her dancing, Makarova (1975), one of the most celebrated classical dancers of the twentieth century replied, “*Even the ears must dance*” (p. 65). This is more than a motto summarizing an artistic approach to a craft—one that emphasizes the thoughtful listening necessary to achieve musicality in dance—it is also an apt description of the overlapping sensory, motor, and psychological processes that underlie the joint activities of music listening and our inclination to move to it.

We have a natural tendency to move to music, and we do so with ease, and without explicit training. This involves the coordinated interplay between different sensory systems that enables us to gain meaningful and multifaceted musical experiences. For children with profound hearing loss, a barrier to music learning stems from a lack of access to salient acoustic cues that form the basis to many musical structures. For an increasing number of profoundly deaf children, this sensory deficit is partially offset by cochlear implants (CIs). These are surgically implanted sensory prostheses that generate hearing-like sensations by means of an electrode array that stimulates the auditory nerve with electrical patterns that code the acoustic features of sound.

### Cochlear implants are effective for speech, but not for music

What is known is that the auditory input of CIs conveys timing information that is within the normal range of hearing listeners (Gfeller and Lansing, 1991; Gfeller et al., 1997). In practice, this timing information can convey acoustic-phonetic features of speech in ideal (i.e., quiet) listening conditions (Remez et al., 1981; Wilson, 2000). Fine structure information necessary for pitch perception, however, is omitted (Shannon et al., 1995) at the expense of this timing information.

Incomplete as this form of electric hearing is, CIs afford the possibility for children who lost their hearing early in life to become good oral communicators, and many children with CIs can attain speech proficiencies that are on par with their hearing peers (Svirsky et al., 2000). In other domains such as music and voice perception, however, such constrained auditory input presents many challenges on child users' listening skills (Gfeller and Lansing, 1991; Stordahl, 2002; Vongpaisal et al., 2012) because they are mainly reliant on timing cues to decode non-speech communication signals. This presents a unique challenge for CI users in their attempts to make sense of sounds where pitch cues are prominent and timing cues are less important. For instance, melody recognition depends critically on pitch cues and less so on timing cues. Consequently, CI children have difficulty recognizing popular folk tunes (Stordahl, 2002). However, they are able to recognize songs at well-above chance levels when the original acoustic cues at the time of learning are presented (Vongpaisal et al., 2006, 2009).

For the purposes of music listening, CI users are able to use available timing cues to detect tempo in music (Kong et al., 2004), to discriminate rhythm (Gfeller and Lansing, 1991; Gfeller et al., 1997), and to recognize familiar songs when original pitch and timing cues are preserved (Volkova et al., 2014). However, it is not known to what extent these timing cues are perceptible to CI users in complex sound scenes, especially when pitch and timbral features co-occur and obscure temporal features of music. Furthermore, most Western music places greater emphasis on melodic detail, with less distinctiveness occurring in the temporal and rhythmic dimension (Fraisse, 1982). Thus, timing-based memory representations form weaker representations of songs in comparison to melodic-based ones (Hébert and Peretz, 1997), making song recognition on the basis of temporal cues a difficult task (White, 1960; Volkova et al., 2014).

Consequently, music listening based exclusively on temporal features presents a unique challenge for CI users and may limit music learning and appreciation to its full capacity, more often in adult recipients with previous hearing experience than child recipients (Fujita and Ito, 1999; Gfeller et al., 2002). Remarkably, many child CI recipients acquire music appreciation demonstrating that their perceptual acuity problems do not deter their enjoyment of music with many incorporating music and dance activities in their daily life (Stordahl, 2002; Mitani et al., 2007; Trehub et al., 2009). Nevertheless, their music learning lags behind that of their hearing peers (Gfeller and Lansing, 1991; Stordahl, 2002). Since the aforementioned limitations are unlikely to be resolved with the current configuration of CI devices, the challenge then becomes one of optimizing the available cues through novel multimodal learning strategies.

### Music and movement go hand in hand: auditory and motor contributions to learning

Much of the research conducted to date on CI children's music perception has focused on assessing their listening-based musical skills (Gfeller and Lansing, 1991; Stordahl, 2002; Gfeller et al., 2005; Vongpaisal et al., 2006, 2009). Not surprisingly, CI children largely underperform their hearing peers in an array of music perception tasks (Gfeller and Lansing, 1991; Vongpaisal et al., 2004). However, such a restricted approach does not consider the role of the other senses and the multiple influences that contribute to the rich and varied musical experiences of listeners in natural settings. Furthermore, the others senses may be especially important in compensating for the restricted auditory input of current devices, thereby providing CI children an alternative route to music. For instance, hearing listeners are propelled to move to music from tapping along to the beat to dancing. The embodiment of music—the integration of actions, or purposeful movements, with sensory information to influence how we learn and think about music—involves overlapping systems that enable sensory-motor interactions to occur (Sevdalis and Keller, 2011). That is, sensory experiences can influence movement to music; while movement, in turn, can influence how music is perceived.

This natural affinity to synchronize or *entrain* to music emerges in early life, and the influence of movement on the perception of musical timing is evident in infancy (Phillips-Silver and Trainor, 2005). Although neurological evidence indicates that auditory and motor systems engage and map onto the same neural structures (Chen et al., 2006), much is still unknown about what this joint activation entails. For instance, do auditory and motor systems work independently and thus contribute something unique to learning, or do these systems depend on each other such that the functioning of one system is integral to the functioning of the other?

Some insight into this process has been gained from observing the auditory-motor performance of musicians and its influences on memory formation. For them, learning was greatest under multimodal conditions that engaged auditory and motor systems jointly in comparison to learning that engaged these modalities independently (Palmer and Meyer, 2000; Brown and Palmer, 2012, 2013). The findings suggest that the coupling of motor and auditory learning enhances encoding of music by creating a greater abstract or gist representation of melodies, provides multiple routes for the retrieval of information, and can provide complementary information beyond that enabled by any individual modality (Palmer and Meyer, 2000; Brown and Palmer, 2012, 2013).

While these findings corroborate what is known about the benefits of coupling action and perception in speech and language learning (MacLeod et al., 2010; MacLeod, 2011), they are the first to contribute to a unified framework on multimodal learning in music. Taken together, these findings are conceptually important and of practical significance to our present research on the musical skills of hearing impaired populations. Our focus on CI children allows us to probe more deeply into how this multimodal framework is affected by hearing loss.

### Music entrainment and music learning in children with hearing loss

While there has been no research to date examining beat entrainment in CI children, there has been only one systematic study that examined musical beat synchronization of adult CI users to music. In comparison to hearing controls who can bounce accurately to different renditions of dance stimuli, Phillips-Silver et al. (2015) found that adult CI users bounced best to simplified drum renditions and synchronized poorly to dance stimuli that contained melodic pitch variations. While the diverse hearing histories of adults, who received their implant later in life, no doubt contribute to the variable tracking of musical tempo, the ultimate demonstration of beat entrainment with CIs would be to observe these skills in child CI users who were either born deaf or prelingually deafened.

There is little research to date examining children's movement in dance and its relationship to the psychological functions involved in music listening. However, recent work by Demir et al. (2014) demonstrating the unique contribution of gestural cues to enhance complex language processing in hearing children, lends a strong basis to our predictions on beat entrainment to enhance music learning in children with hearing loss. They found that teachers who used gestures to highlight verbal input during storytelling, encouraged more complex narratives in children's retelling of these stories in comparison to those who learned through auditory or auditory-visual means without gestures. Furthermore, the advantages of gestures were particularly pronounced in children whose language abilities have been compromised by early brain injury (Demir et al., 2014). By extension, the use of rich multimodal cues that include gestural components could augment auditory and musical skill in our sample of children with hearing loss in educational and rehabilitative contexts.

Taken together, the body of research on multimodal learning provides us with a foundation to explore how the coupling of motor and auditory systems can be used to improve musical and communicative outcomes in children with hearing impairments. From a basic research perspective, the study of children with CIs offers an unparalleled opportunity to study the limits of perception, and the conditions that enable the development of listening and communication skills when hearing is impaired and partially restored. Furthermore, the findings will expand our understanding of how the developing auditory and motor systems adapt to sensory deficits in children, how the integration of auditory and other sensory functions contribute to music learning and auditory capacity in general, and how explicit training can alter the barriers imposed by hearing loss.

In short, the coupling of motor and auditory learning enhances encoding of music, provides multiple routes for the retrieval of information, and can complement or compensate for missing information in an individual modality. These findings are conceptually important and of practical significance for the present study on the musical skills of CI children. In turn, studies on CI children allow us to probe more deeply into how this multimodal framework is affected by sensory impairment.

The aim of the present study was to examine whether dancing and movement during music listening can improve CI children's song learning by enhancing their sensitivity to musical time (e.g., beat, rhythm). We predicted that CI children's learning of songs will be better (as demonstrated by higher accuracy scores) when they dance along to the music in comparison to when they listen to the music only. Purposeful movement that is synchronized to the beat is expected to consolidate the encoding of musical timing information to a greater extent than that achieved by passive listening.

## Materials and methods

### Participants

Ten CI children were initially recruited for the present study. However, one CI participant did not complete the study due to disinterest. Except for one child who was implanted in the right ear only, all were bilateral CI users (see Table 1 for individual details). They used their devices for an average of 4.3 years (*SD* = 1.7), ranging from 2 to 6.7 years. All children received a small toy and gift card as a token of appreciation.

**Table 1:** **Demographic details of individual CI participants**.

**CI**	**Age (Years)**	**Side of implantation**	**Age at implantation (year)**	**CI device**	**Cause of deafness**
1	6.5	LR	3.2	Cochlear	Unknown
2	12.5	LR	6 (R); 5 (L)	Cochlear	Mondini's syndrome
3	4.3	LR	1	Cochlear	Jaundice/Bilirubin
4	5.8	LR	2.5	Advanced Bionics	Unknown
5	9.0	R	7	Cochlear	Unknown
6	4.6	LR	1.2	Cochlear	Unknown
7	7.1	LR	0.9 (R); 3 (L)	Cochlear	Connexin 26
8	4.9	LR	0.9	Cochlear	Connexin 26
9	11.7	LR	5 (R); 6 (L)	Advanced Bionics	Auditory neuropathy

A final sample of nine CI children (*M* = 7.4 years, *SD* = 3.0 years) participated in the study. For the listen and dance training task, kinematic analysis from three CI participants was not possible due to the inability to measure stable movement patterns from their motor behaviors. This included running around the room as a response to music listening (*n* = 1), or limited variability in movement due to shyness (*n* = 2). Therefore, data from seven CI children (CI 1, 4, 5, 6, 7, 8, and 9) were amenable to kinematic analyses. We recruited seven individually age-matched normal hearing controls (named accordingly to their CI matched peer: NH 1, 4, 5, 6, 7, 8, and 9 *M* = 8.0 years, *SD* = 3.4 years), who were within one year of their CI peer's age. We relied on parent reports on the normal hearing status of their child. This was confirmed by the experimenters who observed no difficulties in NH children with the listening demands of the tasks.

All parents provided written consent granting their child's participation, and all children provided verbal assent to participate in the present study. The present study was approved through MacEwan University's policy on the ethical review of research with human participants. It was carried out in full accordance with the ethical standards of the Canadian Tri-Council Policy Statement: Ethical Conduct for Research Involving Humans (TCPS 2).

### Stimuli

The set of stimuli consisted of eight song excerpts (20–30 s in duration) of the pop-rock genre chosen to be unfamiliar to children in the current study. We derived a song list (see Table 2) that was child-friendly and that was likely to be unfamiliar to the young children in this sample. All selections were mid- to up-tempo songs that were contemporary popular music hits from previous decades, and were not in regular rotation on television, radio, or other entertainment media at the time of the study. For each child, three different song excerpts were randomly selected for each learning condition. Prior to testing, parents were asked to confirm whether the songs selected for testing were unfamiliar to their child; in all cases, parents did so.

**Table 2:** **Original pop songs used in the present study**.

**Song title**	**Artist**	**Beat frequency (Hz)**
1. ABC	Jackson 5	1.60
2. Candy girl	Jackson 5	1.67
3. I want you back	Jackson 5	1.65
4. Bad day	Daniel Powter	1.22
5. Pretty Baby	Vanessa Carlton	1.23
6. Sk8er boi	Avril Lavigne	2.35
7. Why	Avril Lavigne	1.68
8. I'm yours	Jason Mraz	1.27

Alternative renditions were generated for a subsequent song recognition task. For the mistuned versions of the excerpts, select notes were shifted by 1–2 semitones using a digital audio pitch-correcting software program (Melodyne, Celemony Software GmbH). Piano versions of the excerpts were generated by an experienced musician who performed and recorded the piano versions of the vocal melody and instrumental beat accompaniment in the original pitch and tempo of the excerpts (See Supplementary Materials). The tempo of each song was measured by a metronome in beats per minute and converted to Hertz. Table 2 lists the song set and the beat frequency of individual songs.

### Procedure

Participants were tested individually and learned unfamiliar pop songs in two training condition: (1) Auditory-only: by passive listening to songs, and (2) Auditory-motor: by listening and dancing to songs. For each training condition, children learned a set of three unfamiliar pop songs that were selected randomly from the set in Table 2. The order of training conditions was counterbalanced for each child.

In the auditory-only condition, children were introduced to the song title—which was depicted by a cartoon image—presented onscreen. The images for the song titles were to be used in a subsequent song recognition task. To listen to the song, the child touched, or clicked on, the image onscreen. While the music played through loudspeakers at comfortable listening levels, (65 dB SPL), the child remained seated in front of a blank computer monitor. Each excerpt was played at least two times; however, the songs could be played as many times upon request. In actuality, each excerpt was played between two to four times. No child requested replays beyond this number, presumably to minimize restlessness or disinterest.

**Figure 1:**
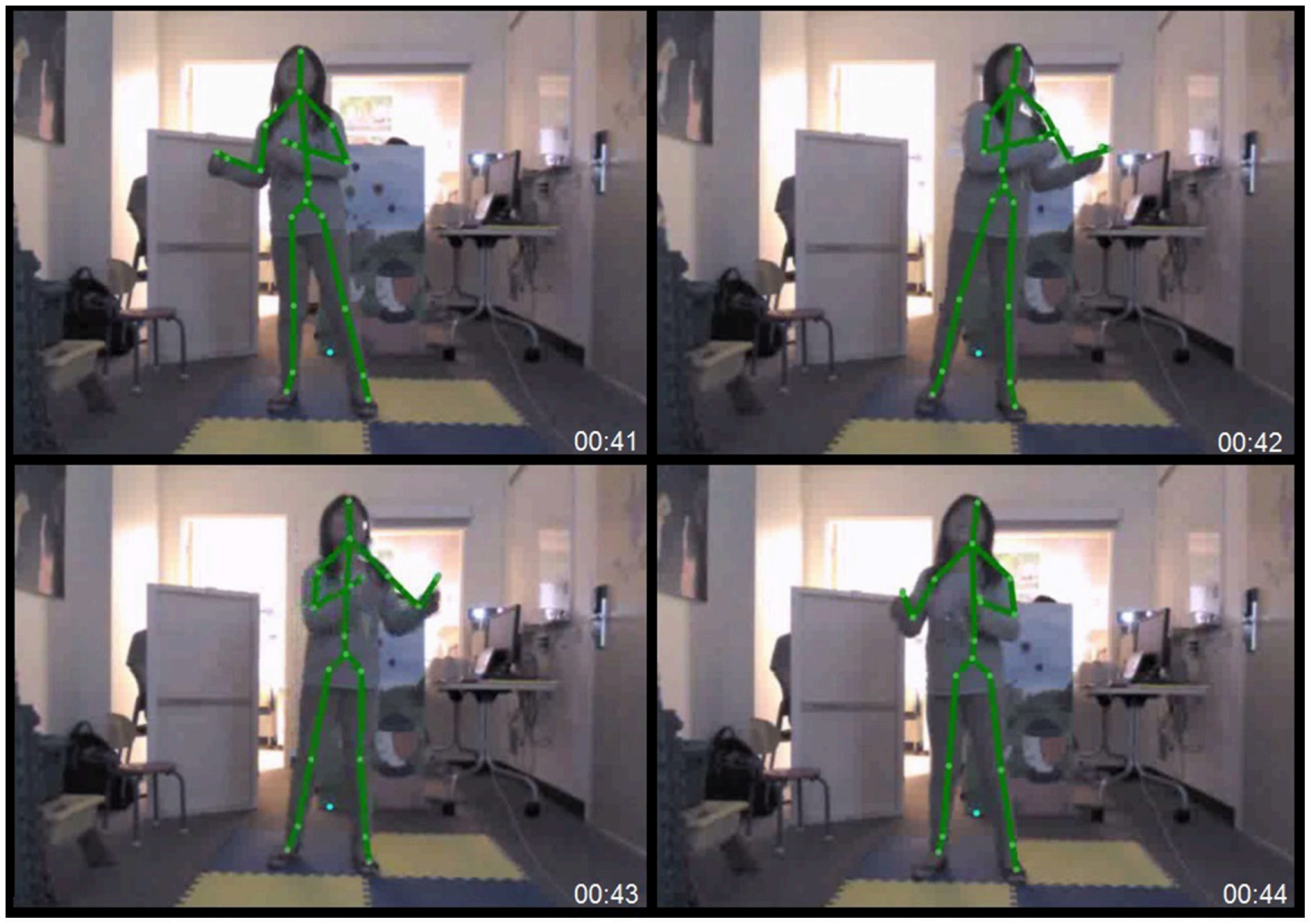
**Motion capture recording (Kinect for Windows) tracking the dancing of a child participant (7 years old)**. Three body indices were chosen for analysis in the present study including the head, distance between hands, and distance between feet.

In the auditory-motor condition, children were presented with a projected point-light image of themselves onscreen (see Figure 1). A three-dimensional motion sensor camera (Microsoft Kinect for Windows), was placed in front of the child (2.1 m from the camera to the center of the dance platform, at a height of 0.76 m from the floor), and was used to capture and record the child's motion. The camera was connected to a laptop computer, which also controlled a multimedia projector (Optoma DW339) that displayed the captured point-light image on a screen (2.1 × 1.2 m, width by height, respectively) positioned in front of the child. This setting enabled the child to view his or her movements mirrored in the point-light image while the music played. Figure 1 shows the joint indices captured from a child's dancing in the auditory-motor learning condition. Twenty body indices were tracked and recorded for kinematic analyses to be conducted offline.

Children were instructed that the point-light image moved along with them, and their task was to dance, or generate movements, as they listened to the music. As with the auditory-only condition, each song excerpt was preceded by the presentation of the song title in the form of a cartoon image displayed onscreen, after which the music played. Each excerpt was played at least two times during which the children were encouraged to dance along to the songs. This was repeated as requested until they felt they could remember the songs. As with the auditory-only condition, no child requested more than four replays for any given excerpt.

Immediately following each training session, children's song learning was assessed in a computerized task that presented the original song excerpts and alternative versions including the mistuned and piano versions. Songs versions were presented in blocks, and the block order was randomized for each participant. Within each block, song excerpts were presented twice (for a total score out of 6) in pseudorandom order with the condition that no excerpt was repeated sequentially. A computer program played song excerpts through loudspeakers and recorded children's selections among three-alternative song title images presented on a touchscreen monitor. Non-contingent feedback was provided in the form of a visually engaging cartoon caricature image that encouraged children to continue.

## Results

Our goal in the present study was to ascertain whether auditory and motor processes engaged in dancing and music listening, in comparison to passive listening, influenced song learning in CI children. Accordingly, our analyses focused on examining children's accuracy in identifying original and alternate versions of songs. We also sought to examine motor entrainment to music by evaluating children's body movement patterns in relation to the beat patterns in music.

### Song recognition task

**Figure 2:**
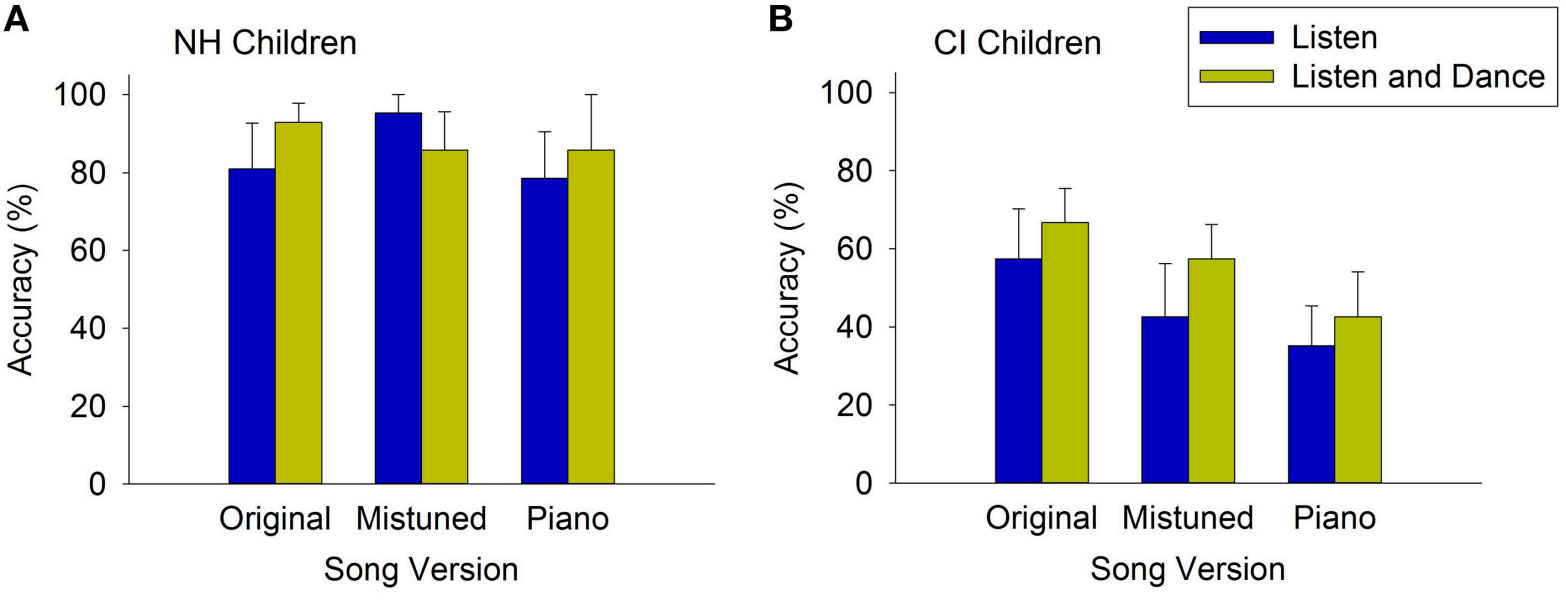
**Song recognition accuracy of NH children (A) and CI children (B) in the original and alternative song renditions in the listen-only (auditory) and listen-and-dance (auditory-motor) training conditions**. Error bars represent standard errors of the mean.

Figure 2A shows that NH children's accuracies across conditions were uniformly high. One-sample *t*-tests confirmed that all six mean scores across learning and song version conditions are not significantly different from perfect accuracy, 100% (*p*s > 0.05). Therefore, any benefit of auditory-motor training would not emerge due to the overall ceiling performance of hearing children in this task.

**Figure 3:**
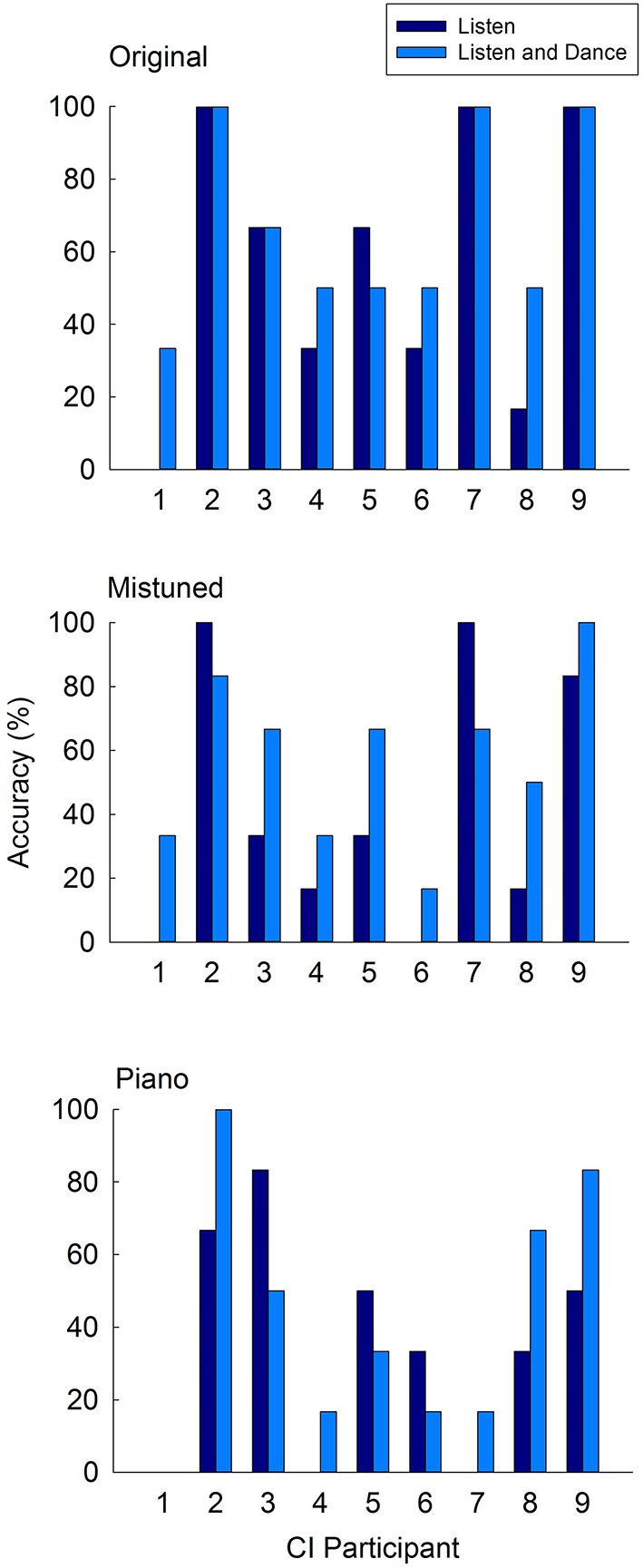
**Individual song accuracy scores for CI participants in listening-only and listen-and-dance learning conditions**.

By comparison, CI children achieved more modest accuracies and showed greater variability in performance across conditions. Figure 2B shows the accuracy scores of CI children across song versions in each training condition, and individual scores are presented in Figure 3. To assess for any order effects in the administration of training conditions, we examined whether there was any difference in the overall mean scores between children who were instructed to listen-only first (*n* = 5), and those who were instructed to listen-and-dance first (*n* = 4). An independent samples *t*-test shows that there was no significant difference (*p* > 0.05) in the overall mean scores that is attributed to training order differences between these groups (*M* = 46.1 and 55.6%; *SD* = 37.6 and 27.7%, listen only first, and listen-and-dance first, respectively).

A two-way within subjects ANOVA was used to examine training (listen-only, listen and dance) and song version (original, mistuned, piano) effects on CI children's recognition accuracy. A main effect of training was found, *F*_(1, 8)_ = 6.28, *p* = 0.037, indicating that CI children's overall accuracy in the listen and dance training (*M* = 55.5%, *SD* = 30.0%) was greater than that in the listen-only condition (*M* = 45.1%, *SD* = 36.5%). In addition, a main effect of song version was found, *F*_(2, 16)_ = 3.93, *p* = 0.041. Paired-sample *t*-tests reveal that CI children's recognition of the original song versions (*M* = 62.0%, *SD* = 32.2%) was more accurate than their recognition of mistuned versions (*M* = 50.0%, *SD* = 34.3), *t*_(17)_ = 3.20, *p* = 0.005. In addition, their accuracy on the original versions was greater than that on piano versions (*M* = 38.9%, *SD* = 31.8%), *t*_(17)_ = 3.08, *p* = 0.007. However, there was no significant difference between their recognition of mistuned and piano versions, *t*_(17)_ = 1.36, *p* = 0.19. The two-way interaction between training and song version was not significant, *F* < 1.

To examine the difference between training conditions more closely, we conducted one-sample *t*-tests to compare the mean scores in each condition against chance performance, where the chance probability is 1/3. In the listen-only training condition, CI children's recognition accuracy of the original versions approached significance, *t*_(8)_ = 1.888, *p* = 0.096. However, their accuracies in the mistuned and piano versions were not significantly different from chance performance, *p*s > 0.05. In the listen and dance training condition, however, CI children scored above chance in the original and mistuned versions (*p*s = 0.005, 0.026), while their accuracies on the piano versions were not different from chance (*p* > 0.05).

Furthermore, inspection of individual accuracies in the original versions (Figure 3) reveals that four children (CI 1, 4, 6, 8) showed gains from listen and dance training. Three other CI children (CI 2, 7, 9) achieved perfect scores that were equal to those achieved by listening only, and only one CI child (CI 5) scored slightly lower following listen and dance training. A clear advantage for listen and dance training, however, was observed in the recognition of mistuned songs. While three CI children (CI 4, 6, 8) showed this advantage across original and mistuned versions, the majority (7 of 9; including CI 1, 3, 4, 5, 6, 8, and 9) performed better following listen and dance training in this version. Scores in the piano versions showed no clear pattern associated with training, presumably due to the less distinctive beat cues in these versions in comparison to the full instrumental versions.

To determine the magnitude of the auditory-motor advantage, we examined the relationship between accuracy scores in the listen-only and listen and dance training. Because the accuracy scores are not normally distributed, a Spearman's correlation was used to examine the association. The analysis revealed a significant and strong positive correlation between accuracy in the listen-only and listen and dance conditions *r*_*s*_ = 0.81, *p* < 0.001. A Cohen's *d* = 0.66 (accounting for learning condition as a within-subjects variable) reveals a medium effect size, indicating that the auditory-motor advantage is of moderate practical significance.

Thus, with short-term exposure to songs as seen in the current study, training involving listening and dancing yielded better than chance performance in versions that contained beat cues in their original instrumentation. The advantage was pronounced in a task that demanded greater transfer of learning, as that occurring in the mistuned versions.

### Analysis of body movement and beat synchronization

While the Kinect motion capture camera tracks and records the movement of 20 body indices (see Figure 1), we focused on three body indices that enabled us to best characterize the full body movement patterns of children in the current experimental set-up. These included movement patterns in the head, distance between hands, and distance between feet.

**Figure 4:**
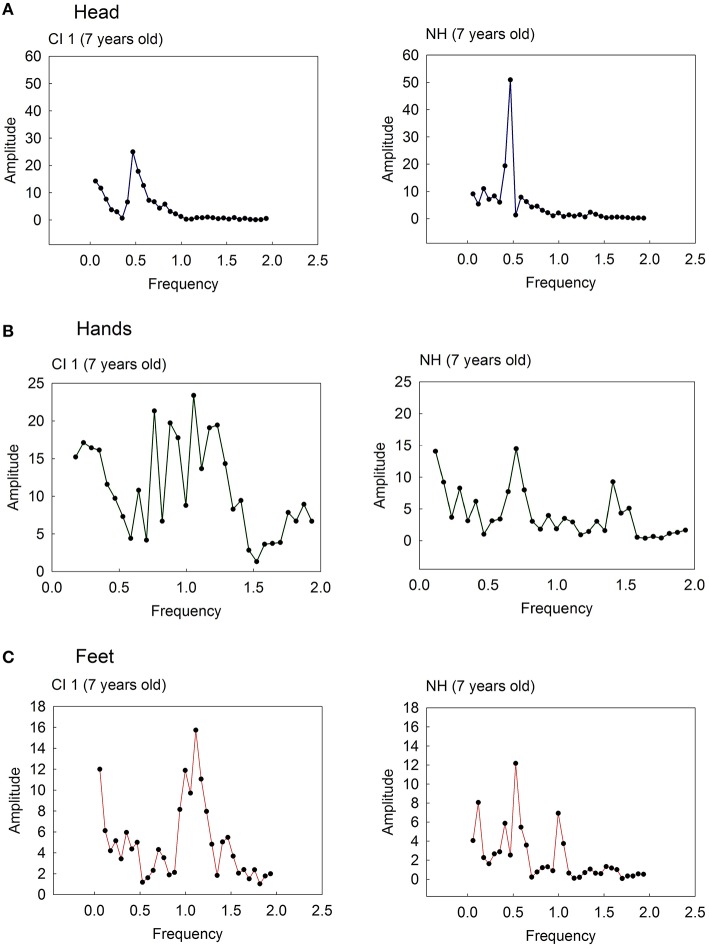
**Frequency distribution of the (A) head, (B) between-hands, and (C) between-feet movements of a child with cochlear implants (CI, 7 years old) and an age-matched normal hearing (NH) child**.

To assess whether children moved in synchrony to the beat of songs, we examined whether the body movement frequencies matched the beat frequencies of the songs. Since children's movement frequencies may vary with song, and may vary according to idiosyncratic movement tendencies, we examined body synchrony at four related beat frequencies according to the following ratios: 0.25:1, 0.5:1, 1:1, and 2:1. For each trial, body movement variability (for head, between-hands, between-feet distances) was computed as the difference between the observed body movement frequency and the expected beat frequency of the song. Figure 4 show the movement frequency distribution of the head, between-hands, and between-feet distances of a 7-year old child and age-match control for the same song. The data were submitted to a Fast Fourier Analysis to extract the dominant movement frequency. As can be seen, a dominant frequency can be extracted from the child's movement patterns (e.g., head), and in some cases, more than one dominant frequency may emerge. This often corresponds to a complex movement sequence comprising more than one movement component. For instance, two dominant frequencies in the NH child's frequency distribution of hand movements correspond to a periodic arm swing and hand shake as part of a single movement sequence to a beat (see Figure 4B). In such cases, we included up to two dominant frequencies in the computation of the average movement frequency for a body index (see Table 3).

**Table 3:** **Mean frequency of head, between-hands, between-feet movements for individual CI participants and age-matched hearing controls**.

**Participant**	**Head (Hz)**		**Hands (Hz)**		**Feet (Hz)**	
	***M***	***SD***	***M***	***SD***	***M***	***SD***
CI 1	0.63	0.09	1.17	0	1.09	0.27
CI 4	0.98	0.17	–	–	–	–
CI 5	0.79	0.21	0.92	0.38	–	–
CI 6	0.47	0.16	–	–	–	–
CI 7	0.52	0.08	0.70	0.10	–	–
CI 8	–	–	0.87	0.23	–	–
CI 9	0.68	0.29	0.48	0	0.91	0.37
NH 1	0.41	0.13	0.64	0.15	0.96	0.31
NH 4	0.96	0.20	1.23	0.76	1.32	0.37
NH 5	0.52	0.12	–	–	–	–
NH 6	0.43	0.23	–	–	–	–
NH 7	0.59	0.06	1.02	0.31	0.86	0.24
NH 8	0.32	0.08	0.64	0.12	0.95	0
NH 9	0.49	0.12	–	–	1.17	0

**Figure 5:**
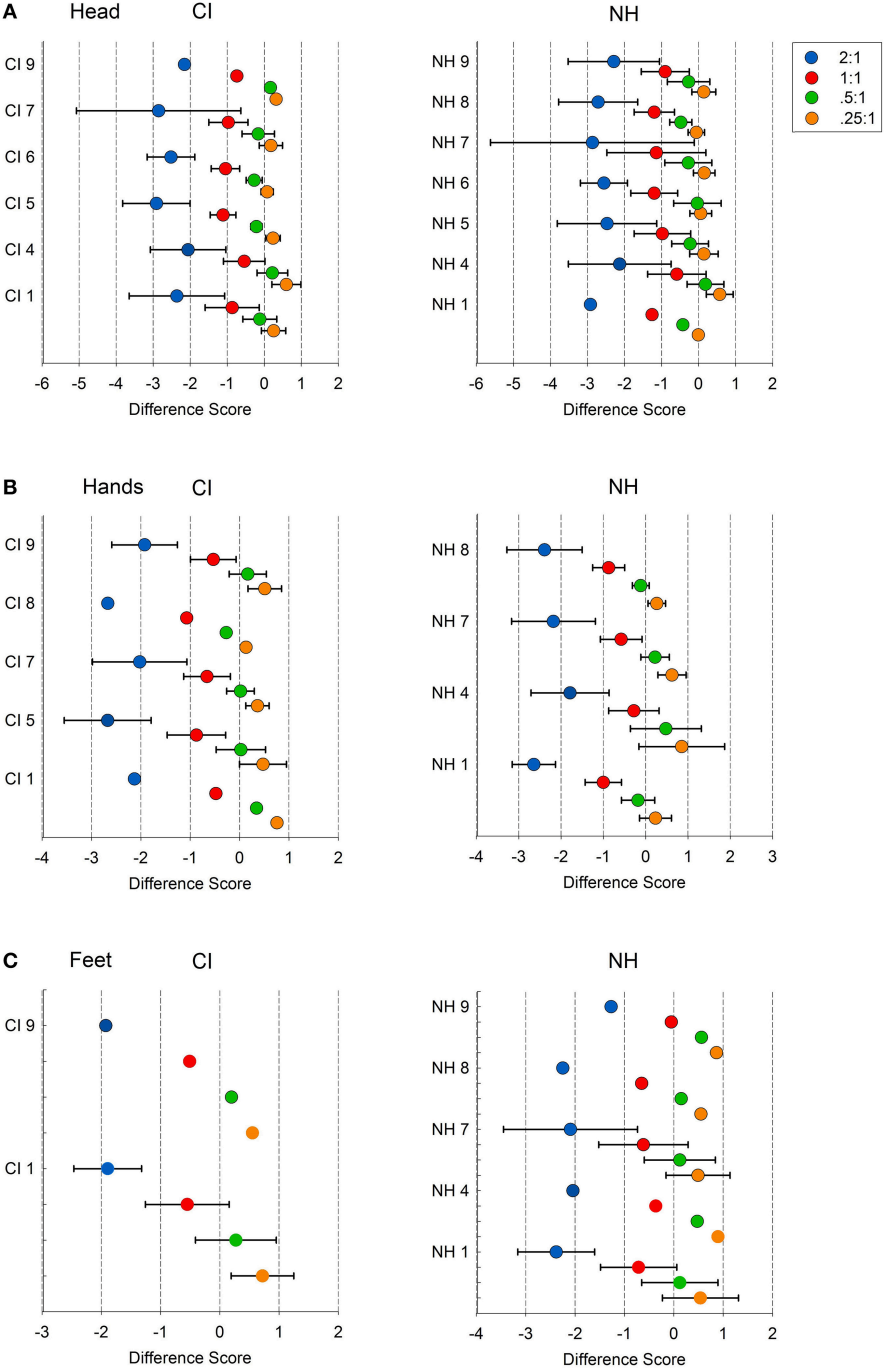
**Mean difference scores for individual CI children and age-match controls at four beat frequency levels (2:1, 1:1, 0.5:1, and 0.25:1) and for three body indices: (A) head, (B) between-hands, and (C) between-feet**. Difference scores are averaged across songs, and are computed from the difference between body movement frequency and song beat frequency. Error bars indicate 95% confidence intervals. Difference scores closer to zero indicate good beat synchrony.

To derive a global measure of body movement variability at each beat frequency level, the average frequency across songs was calculated for each body index. Figure 5 reports the mean movement frequencies for the head, hands, and feet, and associated two-sided 95% confidence intervals, generated by each child. One-sample *t*-tests (2-tailed) on these means were conducted to determine whether the movement variability at each beat frequency level differed from zero. Good beat synchronization occurs when there is a close match (i.e., no significant difference) between the observed body movement frequency and the actual beat frequency level of the song. These analyses were possible when at least three movement frequencies were extracted across all song samples.

When there was an insufficient number of samples to conduct meaningful significance testing, the means were simply plotted (e.g., mean head and hand frequencies for CI 9). As can be seen, for both CI and NH children's movements, confidence intervals most often included zero for the 0.5:1 and 0.25:1 beat structures levels of songs. That is, both groups of children tended to produce body movements that are synchronized to every second beat or every fourth beat in a song, respectively. Inspection of this figure also reveals that fewer children generated periodic movements with their hands and feet.

One notable observation is the occurrence of synchronized body movements to the 1:1 beat level by individual NH children (head: NH 4 and 7; hands: NH 4; and feet: NH 1 and 7) and CI children (hands: CI 4; feet: CI 1). This likely reflects a greater tendency to generate more complex body movements with individual components that are synchronized to more than one beat structure in songs. In short, kinematic analyses indicate that most NH and CI children synchronize to every second and fourth beat in songs, with some generating movement components that synchronize to every beat.

## Discussion

The aim of the present study was to examine whether learning that engages auditory-motor processing during listening leads to better song knowledge than learning that engages auditory processes only. We found that, within the short time span of the present study, dancing to music had an impact on CI children's song learning, as shown by greater memory for songs in a follow-up identification task. This advantage was qualified by a medium effect size (Cohen's *d* = 0.66) indicating that auditory-motor processing in active music listening with dance is of practical importance. By comparison, hearing children performed at ceiling levels in remembering songs learned when dancing and when listening passively to music. Any potential gains from auditory-motor learning were likely masked due to the ease with which hearing children could remember music in the current task.

Although CI children were considerably less accurate than their hearing peers, learning conditions that engaged auditory and motor skills enabled them to identify songs at above chance levels in the original and mistuned transformations. In contrast, their identification of the piano versions was at chance level. This advantage suggests that motor responses to music are most effective in consolidating musical representations in versions that retain the original percussive beats.

We observed that the transfer of learning from listening and dance training was best seen in the mistuned condition—an unfamiliar version that retained the original instrumentation of the percussive beats. Their lower accuracies in the mistuned version, in comparison to the original version, suggest that the original spectral information was important for song recognition as distortions from our pitch shifting manipulation disrupted performance. Nevertheless, it is exceptional that they succeeded in achieving scores above chance levels in mistuned versions when engaged in listening and dancing, while they were unable to do so following training that involved passive listening.

The observed advantage is likely conservative given the short-term and self-determined lengths of exposure to songs in the training session. These margins could be increased over longer training periods and greater duration of exposure to stimuli. In addition, providing children greater structure, or guided direction in generating movements to specific timing structures, could go further in improving beat synchronization and song learning.

To further understand children's motor response to music, we examined whether their dance movements synchronized to the temporal structures in songs. Because the main objective of the present study was to examine children's natural entrainment to the beat, no attempts to constrain or choreograph children's musical movements were made of any kind. What emerged was a picture of children's implicit interpretation of timing features in music, and their natural movement patterns toward them. The majority of children generated improvisational expressive gestures that corresponded with the timing structures in songs.

Kinematic analyses of body movements showed that CI children entrained most frequently to every second or fourth beat in songs, indicating that they can hear and generate a synchronized motor response to temporal structures in music. Furthermore, an inspection of patterns across groups reveals that CI children attuned to key timing features in ways that appear qualitatively similar to those of hearing peers. This finding is consistent with the observations reported by Phillips-Silver et al. (2015) showing that adult CI users entrain to the beat of Latin Meringue music as well as hearing controls; however, for our young sample of CI children, there is no evidence that the complex spectral variations in pop songs interfered with their ability to dance to the beat. This may be due, in part, to the benefits of greater adaptation to electrical auditory input by children, and also to the heightened salience of stereotyped beats in mainstream popular dance music. Taken together, our findings suggest that partial hearing restoration with CIs can enable the development of auditory-motor circuits that support synchronized dance movements to music, which may be underpinned by mirror neuron systems that integrate motor-auditory-visual inputs (Le Bel et al., 2009).

Due to the naturalistic conditions of the study, the results represent the variable and individual movement tendencies of children to music in everyday listening conditions. Some children demonstrated a greater range of full-body movements to music, while other children tended to generate expressive head movements only. Prior to the study, none of the CI children received any formal dance or music lessons, therefore the greater variability in body movements across head, hands, and feet observed in some children is not attributed to any training advantages, and likely reflects individual differences in the production of expressive movement. Future research could determine whether explicit training, or structured learning tasks focusing on music and motor entrainment, could lead to further improvements in temporal pattern processing or musical knowledge in general.

Although it is not possible to determine any systematic effects of demographic variables or device characteristics on performance outcomes in this small sample, inspection across individual results (Figure 3) reveals that the highest song identification accuracies across versions were achieved by the oldest CI children (CI 2 and CI 9). While both of these children were late and sequential bilateral implantees, respectively, (with the latter possibly receiving delayed implantation as a result of auditory neuropathy) their advantage likely stemmed from longer duration of device use and more advanced general cognitive ability than their younger peers. It is also noteworthy that the participant (CI 1) who had the most difficulty with song recognition across versions was among the younger CI participants (CI 3, 4, 6, and 8) in this group (median age 6.5 year old). Furthermore, among these younger CI users, this participant (CI 1) was the oldest at age of implantation despite having similar length of device use. By contrast, all other younger CI participants received their implants at < 3 years of age. Thus, the participant's younger age, in combination with more advanced age at implantation, could underlie the observed poorer performance in this task relative to other CI children in our sample. Finally, it is also notable the only unilateral implantee (CI 5) displayed no particular disadvantage in this task as a result of single-sided CI input, scoring within the range of bilateral implantees.

To what can we attribute CI children's greater success in song learning when listening and dancing to music? Auditory and motor processes engaged simultaneously in music listening can promote the encoding of timing redundancies in music via rich multimodal representations of musical structure. This entrainment to music can generate heightened attention to musical features rendering them more salient for learning and memory in comparison to processing that occurs in one modality alone (Bahrick et al., 2002). This is supported by evidence indicating that neural responses to rhythms are enhanced following training that couples hand tapping movements to auditory rhythm processing (Chemin et al., 2014).

While multimodal processing involving visual-motor (Horn et al., 2007) and fine motor skills (Horn et al., 2006) have been linked with language outcomes in CI children, the present findings are an important first step toward understanding the basic auditory-motor contributions to music learning in these children. Our goal of linking motor behavior and perception in CI children's music learning may have broader implications in facilitating their learning in a range of non-musical domains. This is based on a growing body of research showing that the same auditory and motor skills engaged in music could transfer to the language domain by increasing children's sensitivity to acoustic speech features (Tierney and Kraus, 2013). In short, our findings indicate that learning strategies that recruit complementary multimodal information and capitalize on entrainment, can enhance learning and memory for music. Accordingly, this sets an important precedent, in future CI research, to examine the possible transfer of multimodal music learning to other domains that depend on good auditory capacity and listening skills.

## Author contributions

TV was primarily responsible for the conception and design of the study. All authors undertook the major task of data acquisition. All authors contributed to the analysis and interpretation of the data, as well as to the preparation and final approval of the manuscript. Finally, all authors are accountable for all aspects of the present study.

### Conflict of interest statement

The authors declare that the research was conducted in the absence of any commercial or financial relationships that could be construed as a potential conflict of interest.
